# Author Correction: Entry of spores into intestinal epithelial cells contributes to recurrence of *Clostridioides difficile* infection

**DOI:** 10.1038/s41467-022-30283-x

**Published:** 2022-04-29

**Authors:** Pablo Castro-Córdova, Paola Mora-Uribe, Rodrigo Reyes-Ramírez, Glenda Cofré-Araneda, Josué Orozco-Aguilar, Christian Brito-Silva, María José Mendoza-León, Sarah A. Kuehne, Nigel P. Minton, Marjorie Pizarro-Guajardo, Daniel Paredes-Sabja

**Affiliations:** 1 Microbiota-Host Interactions and Clostridia Research Group, Facultad de Ciencias de la Vida, Universidad Andrés Bello, Santiago, Chile; 2 ANID – Millennium Science Initiative Program - Millennium Nucleus in the Biology of the Intestinal Microbiota, Santiago, Chile; 3 School of Dentistry and Institute for Microbiology and Infection, University of Birmingham, Birmingham, UK; 4 BBSRC/EPSRC Synthetic Biology Research Centre, School of Life Sciences, Centre for Biomolecular Sciences, The University of Nottingham, Nottingham, UK; 5 Department of Biology, Texas A&M University, College Station, TX USA

**Keywords:** Bacterial pathogenesis, Cellular microbiology, Pathogens, Infection

Correction to: *Nature Communications* 10.1038/s41467-021-21355-5, published online 18 February 2021.

The original version of this Article contained an error in the Methods, section ‘Spore preparation’, which incorrectly read ‘6.3% weight vol^−1^ (BD, USA), 0.35% weight vol^−1^ protease peptone’. The correct version replaces this text with ‘6.3% weight vol^−1^ bacto peptone (BD, USA), 0.35% weight vol^−1^ proteose peptone’.

In the Methods, the incorrect supplier and catalogue number for an antibody was listed. The sentence ‘phalloidin Alexa-Fluor 568 (#ab176753 Abcam, USA)’ should read ‘phalloidin Alexa-Fluor 568 (#A12380 Thermo Fisher, USA)’.

The Methods, in the section ‘Colonic and ileal loop assay ’, originally incorrectly read ‘To evaluate the effect of nystatin or RGD peptide in *C. difficile* spore internalization, mice were treated with 17,000 UI kg^−1^ nystatin (*n* = 4) 24 h before the surgery. In the loop, as control, mice were treated with 0.9% NaCl (saline; *n* = 4) then, ligated loops were injected with 3 × 10 *C. difficile* R20291. In the case of RGD, ligated loops were injected with 250 nmol of RGD peptide (*n* = 4)’. The correct version replaces this text with ‘To evaluate the effect of nystatin and RGD peptide in *C. difficile* spore internalization in vivo, 24 h prior to surgery, mice (*n* = 4) were treated with nystatin (17,000 IU kg^−1^) in 100 µL of DPBS by oral gavage; control mice (*n* = 8; 4 for control and 4 for RGD treatment) where treated with 100 µL of DPBS by oral gavage. On the day of surgery, ileal and colonic ligated loops of control mice (*n* = 4) were injected with 100 µL of DPBS containing 3 × 10^8^ *C. difficile* R20291 spores; ileal and colonic ligated loops of nystatin-treated mice (*n* = 4) were injected with 100 µL of DPBS containing 3 × 10^8^ *C. difficile* R20291 spores and 340 IU (17,000 IU kg^−1^) nystatin; ileal and colonic ligated loops of RGD-treated mice (*n* = 4) were injected with 100 µL of DPBS containing 3 × 10^8^ *C. difficile* R20291 spores and 86.6 µg (250 nM) of RGD peptide. DPBS was used to resuspend nystatin and RGD peptides because it rendered higher solubility than saline solution (0.9% weight vol^−1^ NaCl)’.

The Methods, in the section ‘Quantification of *C. difficile* spores from feces and colon of mice’, originally incorrectly read ‘1.5% weight vol^−1^ (BD, USA; TCCFA plates)’. The correct version replaces this text with ‘1.5% weight vol^−1^ agar (BD, USA) (TCCFA plates)’.

The original version of this Article incorrectly cited ‘Bakken, T. L. & Sageng, H. Mental health nursing of adults with intellectual disabilities and mental illness: a review of empirical studies 1994–2013. *Arch. Psychiatr. Nurs*. **30**, 286–291 (2016)’ as Ref. 4. The correct version replaces this reference with ‘Evans, C. T. & Safdar, N. Current trends in the epidemiology and outcomes of *Clostridium difficile* infection. *Clin. Infect. Dis*. **60** (Suppl 2), S66-71 (2015)’.

These errors have been corrected in the PDF and HTML versions of the Article.

