# Supplementary material for: Entry of spores into intestinal epithelial cells contributes to recurrence of Clostridioides difficile infection
Source: Nat Commun. 2021 Feb 18;12:1140. doi: 10.1038/s41467-021-21355-5 (PMC7893008; doi:10.1038/s41467-021-21355-5)
Supplement: Supplementary file 1 — Supplementary Information [file 41467_2021_21355_MOESM1_ESM.pdf]

Supplementary Information

Entry of spores into intestinal epithelial cells contributes to recurrence of *Clostridioides difficile* infection

Pablo Castro-Córdova, Paola Mora-Uribe, Rodrigo Reyes-Ramírez, Glenda Cofré-Araneda, Josué Orozco-Aguilar, Christian Brito-Silva, María José Mendoza-León, Sarah A. Kuehne, Nigel P., Minton, Marjorie Pizarro-Guajardo and Daniel Paredes-Sabja.

Table of contents:

Supplementary Figures 1 – 18 ……………………………………………........….2

Supplementary Tables 1 – 2 …………………………………………………….….22

Supplementary References……………………………………………………….25

Supplementary Figures

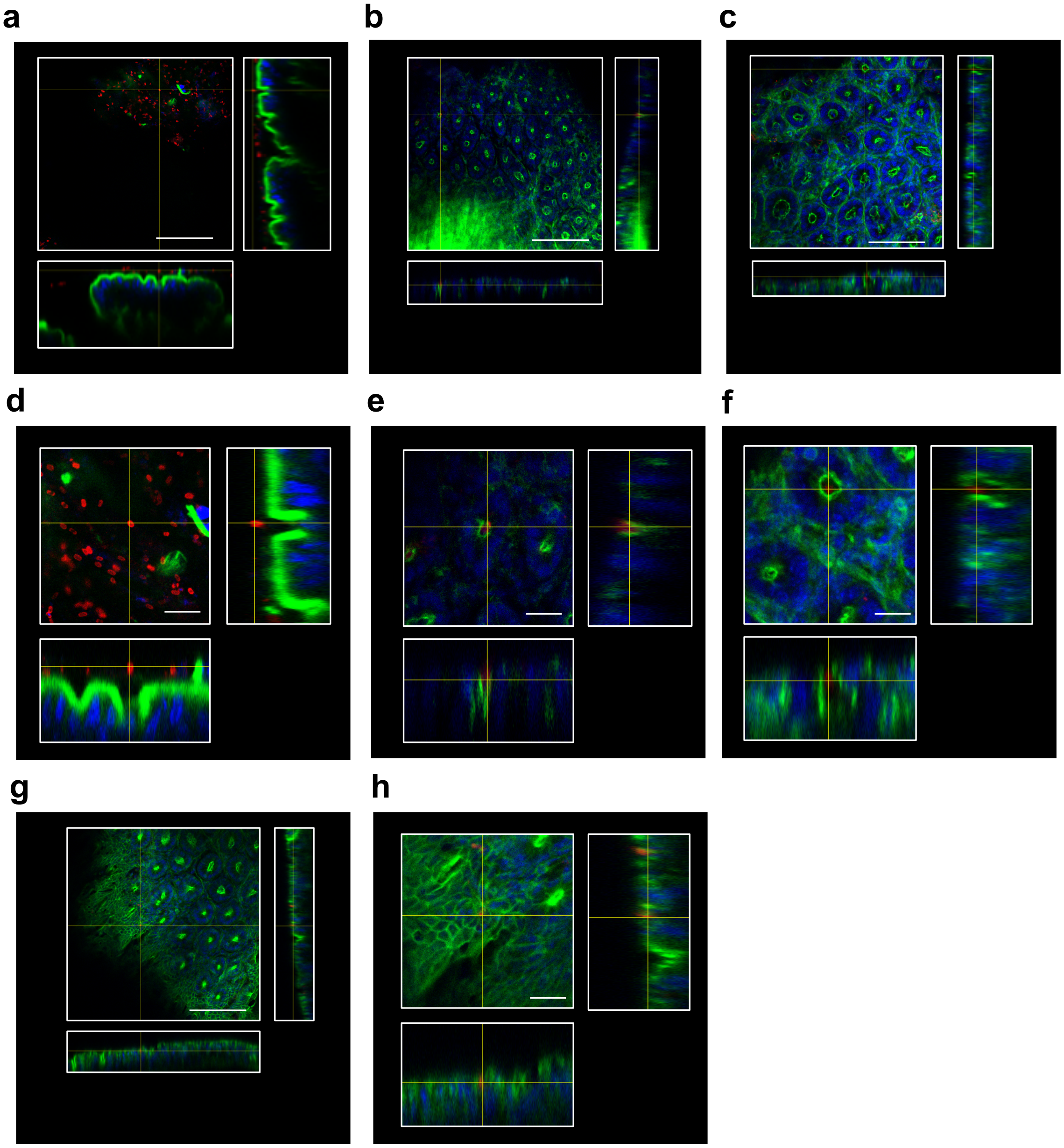

Supplementary Figure 1. Adherence of *C. difficile* spores to the colonic mucosa. Representative confocal micrographs of fixed whole-mount colonic tissue of mice. Panels **a**–**c**, **g** micrographs of colon of C57BL/6 mice infected in a colonic loop model with 5×10^8 *C. difficile* R20291 spores for 5 h. *C. difficile* spores are shown in red, F-actin is shown in green, and nuclei in blue (fluorophores colors were digitally reassigned for a better representation). Panels **d**–**f**, **h** are a magnification of panels **a**–**c**, **g** respectively.

Highlighted are *C. difficile* spores close to the apical membrane of cells. Micrographs are representative of 3 independent mice. Bars **a**−**c**, **g** 50 μm, **d**−**f**, **h** 10 μm.

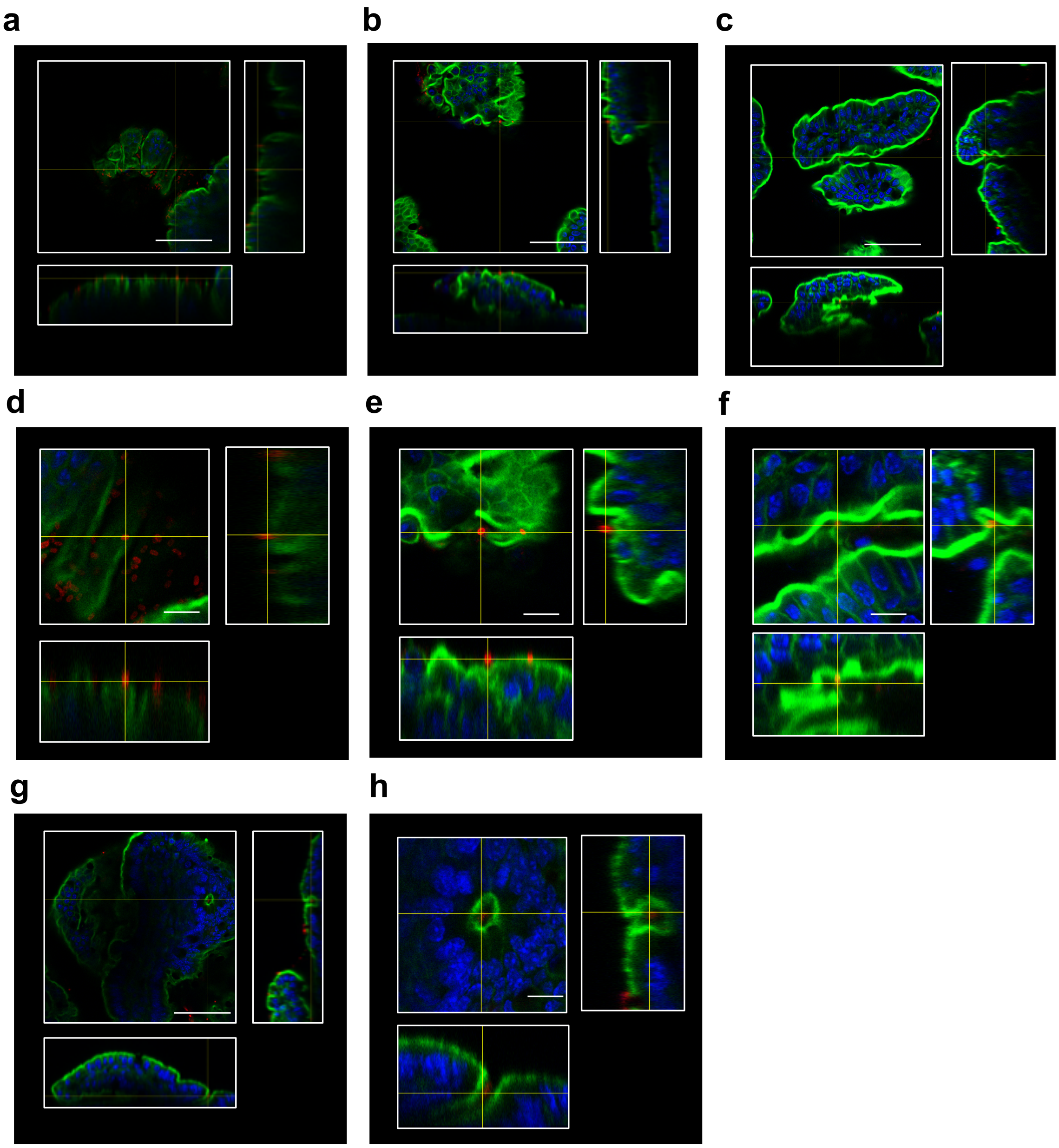

Supplementary Figure 2. Adherence of *C. difficile* spores to the intestinal mucosa. *z*-plane and orthogonal view of confocal micrographs of fixed whole-mount colonic tissue of mice. Panels **a**−**c**, **g** micrographs of ileum mucosa of C57BL/6 mice infected in an ileal loop model with 5×10^8 spores *C. difficile* R20291 spores for 5 h. *C. difficile* spores are shown in red, F-actin is shown in green and nuclei in blue (fluorophores colors were digitally reassigned for a better representation). Panels **d**−**f**, **h** are a magnification of panels **a**−**c**, **g** respectively. Highlighted are *C. difficile* spores close to the apical membrane of cells. Micrographs are representative of 3 independent mice. Bars **a**−**c**, **g**, 50 μm, **d**−**f**, **h**, 10 μm.

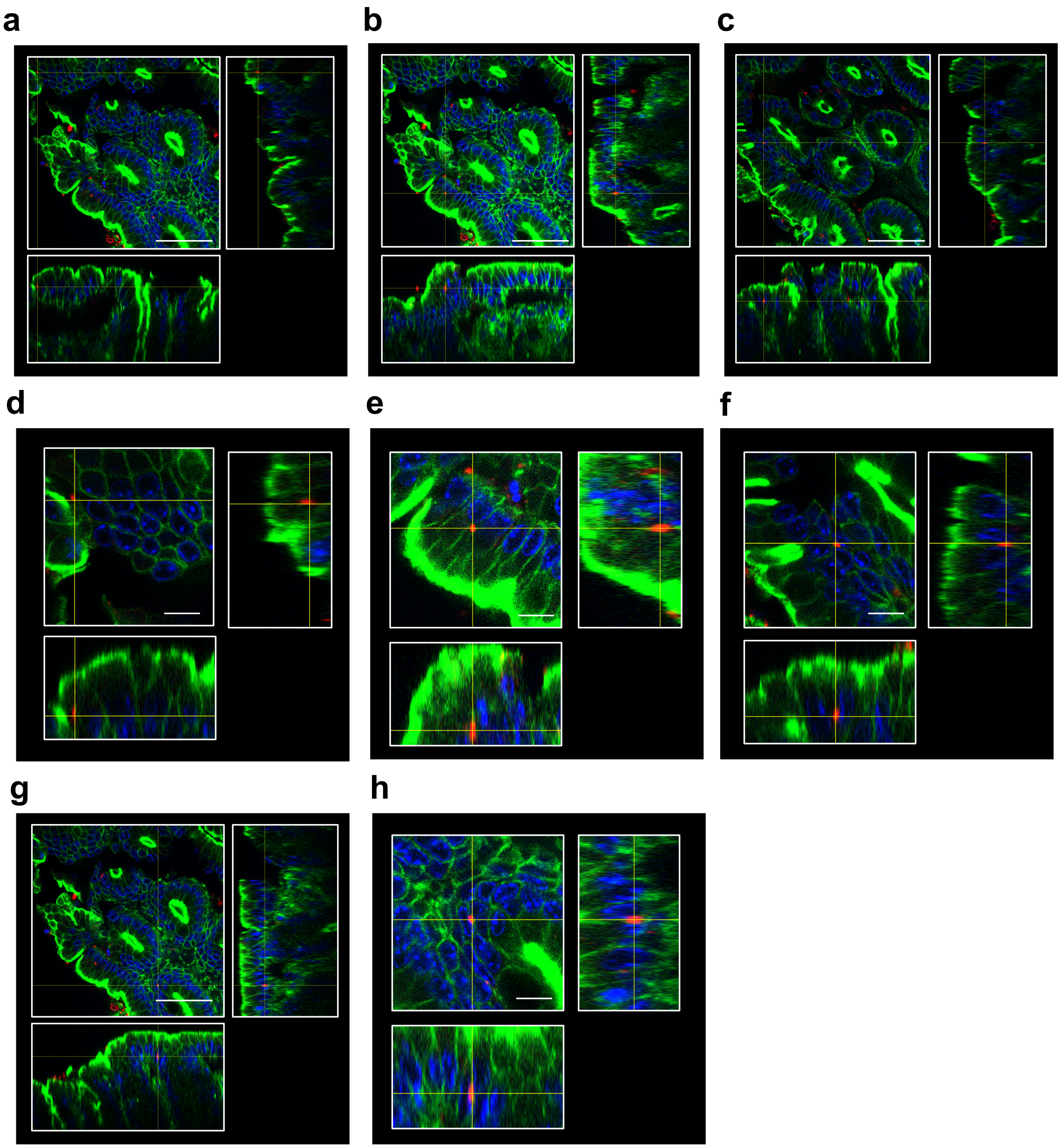

Supplementary Figure 3. Internalization of *C. difficile* spores to the colonic mucosa *in vivo*. *z*-plane and orthogonal view of confocal micrographs of fixed whole-mount colonic tissue of mice. Panels **a**−**c**, **g**, Confocal micrographs of the colonic mucosa of C57BL/6 mice infected in a colonic loop model with 5 × 10^8 *C. difficile* R20291 spores for 5 h. *C. difficile* spores are shown in red, F-actin is shown in green, and nuclei in blue (fluorophores colors were digitally reassigned for a better representation). Panels **d**−**f**, **h** are a magnification of panels **a**−**c**, **g**, respectively. Highlighted are *C. difficile* spores close the apical membrane of cells. Micrographs are representative of 3 independent mice. Bars **a**−**c**, **g**, 50 μm, **d**−**f**, **h**, 10 μm.

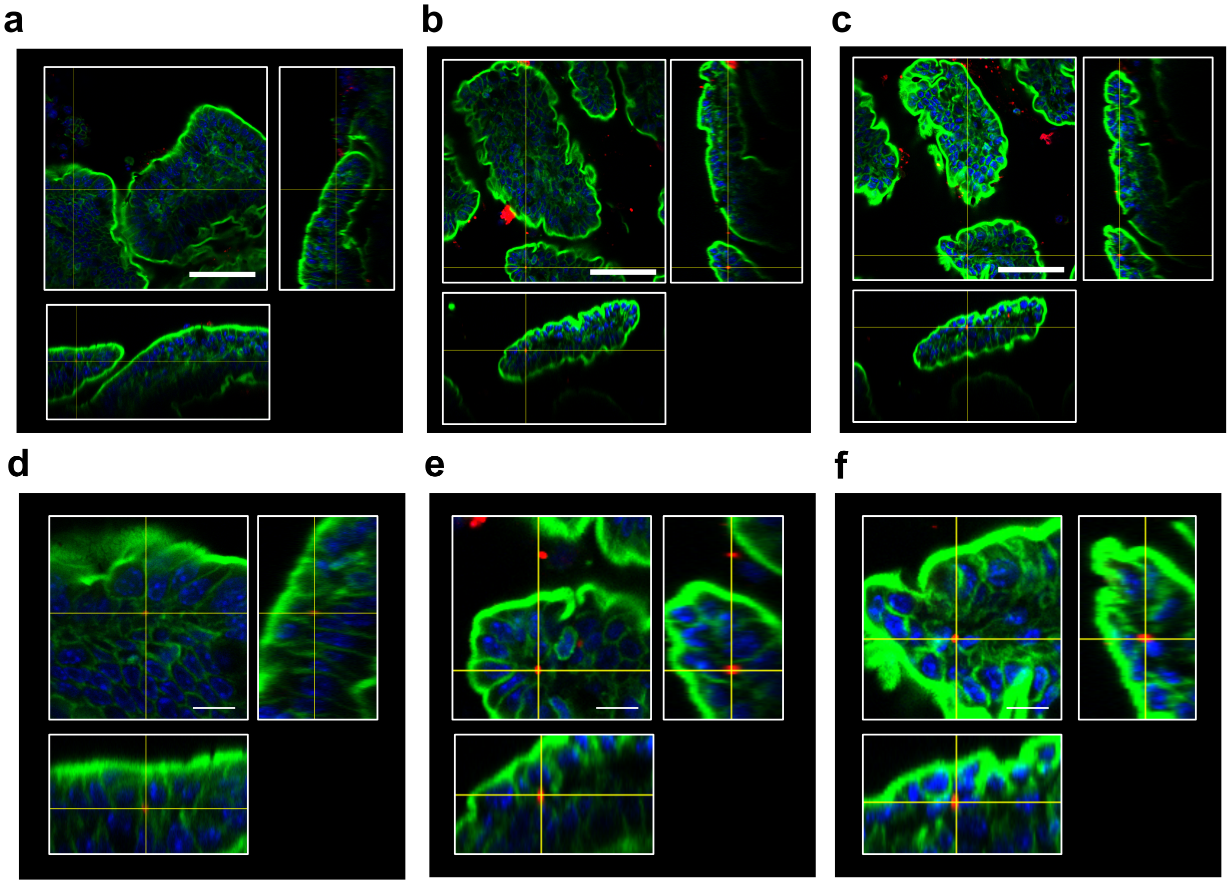

Supplementary Figure 4. Internalization of *C. difficile* spores into the intestinal mucosa. *z*-plane and orthogonal view of confocal micrographs of fixed whole-mount colonic tissue of mice. Panels **a**–**c** micrographs of the ileum mucosa of C57BL/6 mice infected in an ileal loop model with 5×10^8 spores *C. difficile* R20291 spores for 5 h. *C. difficile* spores are shown in red, F-actin is shown in green and nuclei in blue (fluorophores colors were digitally reassigned for a better representation). Panels **d**–**f**, **h** are magnification of panels **a**–**c**, **g** respectively. Highlighted are *C. difficile* spores close the apical membrane of cells. Micrographs are representative of 3 independent mice. Bars **a**–**c**, **g** 50 μm, **d**–**f**, **h** 10 μm.

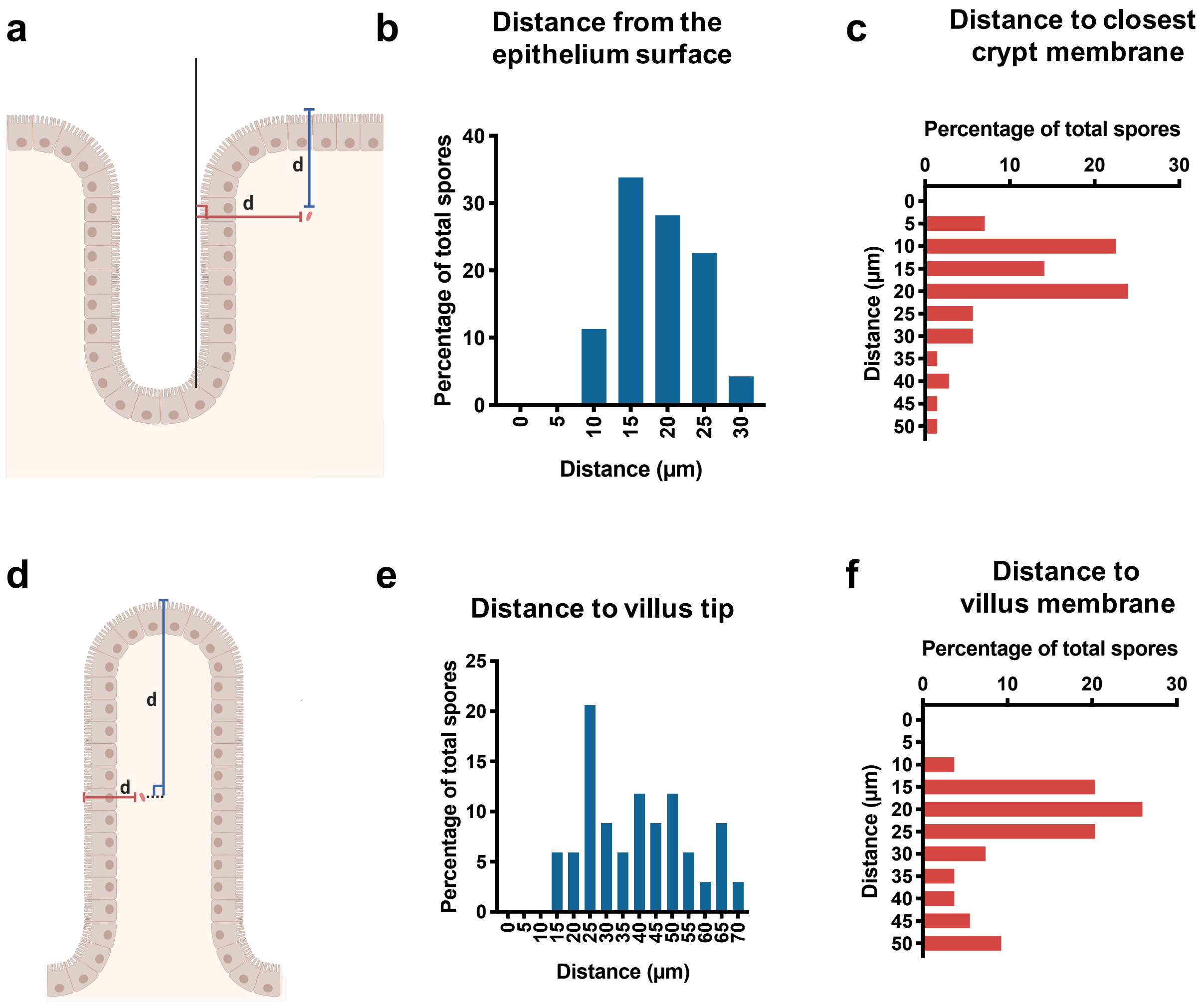

Supplementary Figure 5. Distribution of internalized *C. difficile* spore in colonic and in ileum mucosa. **a** Schematics representation of the method applied for measurements of distances of internalized spores in the ileum mucosa. Distribution of the distance of internalized *C. difficile* spores to the **b** villus tip or to **c** the villus membrane. **d** Schematics representation of the method applied for measurements of distances of internalized spores in the colonic mucosa. **e** Distribution of the distance of internalized *C. difficile* spores to the colonic epithelium surface or **f** to the closest crypt axis. In the ileum, distance from 34 internalized *C. difficile* spores to the villus tip and the distance of 54 internalized *C. difficile* spores to the villus membrane is shown. In the colon, the distance of 61 internalized *C. difficile* spores was evaluated. **a**, **d** Created with BioRender.com.

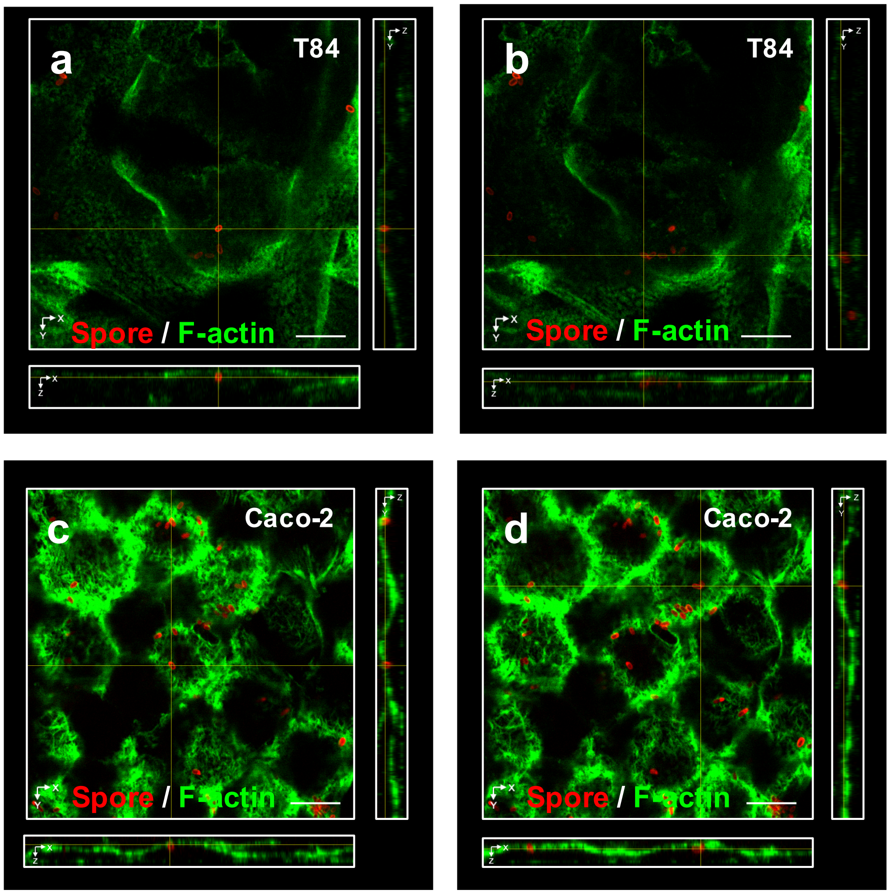

Supplementary Figure 6. Confocal microscopy of internalized *C. difficile* spores into intestinal epithelial cells *in vitro*. *z*-plane and orthogonal view of confocal micrographs of **a**, **b** polarized T84 cells infected with NHS-treated R20291 spores for 5 h. **c**, **d** Intestinal epithelial Caco-2 cells infected with NHS-treated *C. difficile* R20291 spores. *C. difficile* spores are shown in red, F-actin is shown in green, (fluorophores colors were digitally reassigned for a better representation). The images are representative of 2 independent experiments. Scale bar, 10 µm.

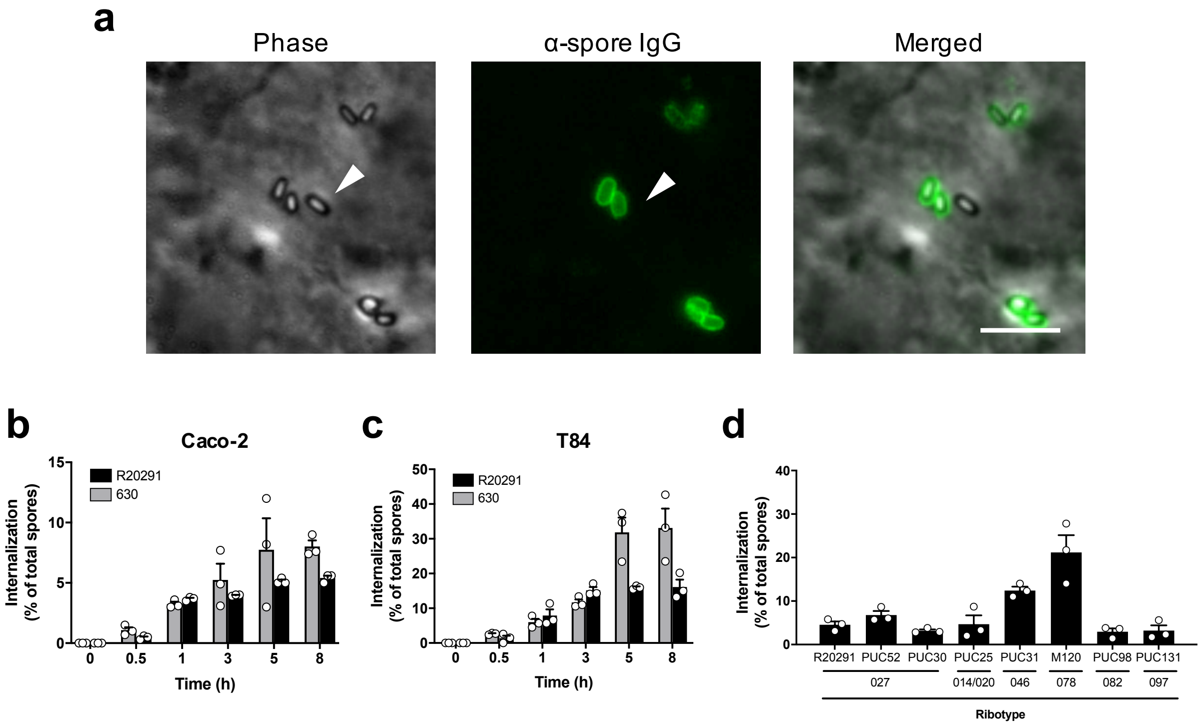

Supplementary Figure 7. Internalized spores are not stained with anti-*C. difficile* spore goat serum in non-permeabilized cells and serum increases the internalization into Caco-2 cells. **a**, Representation of the method used to identify internalized *C. difficile* spores. In infected cells without permeabilization, spores were immunodetected as described in methods, and bright spores that were not detected with fluorescent-labeled antibodies were considered internalized. Dynamic of entry of spore *C. difficile* R20291 and 630 pre-incubated with FBS in infected **b** Caco-2, and **c** T84 cells. **d** Internalization of *C. difficile* spores of clinical isolates of various ribotypes into Caco-2 cells. Scale bar 5µm. In bars, each dot represents one independent well from three independent experiments. Error bars indicate the mean ± S.E.M.

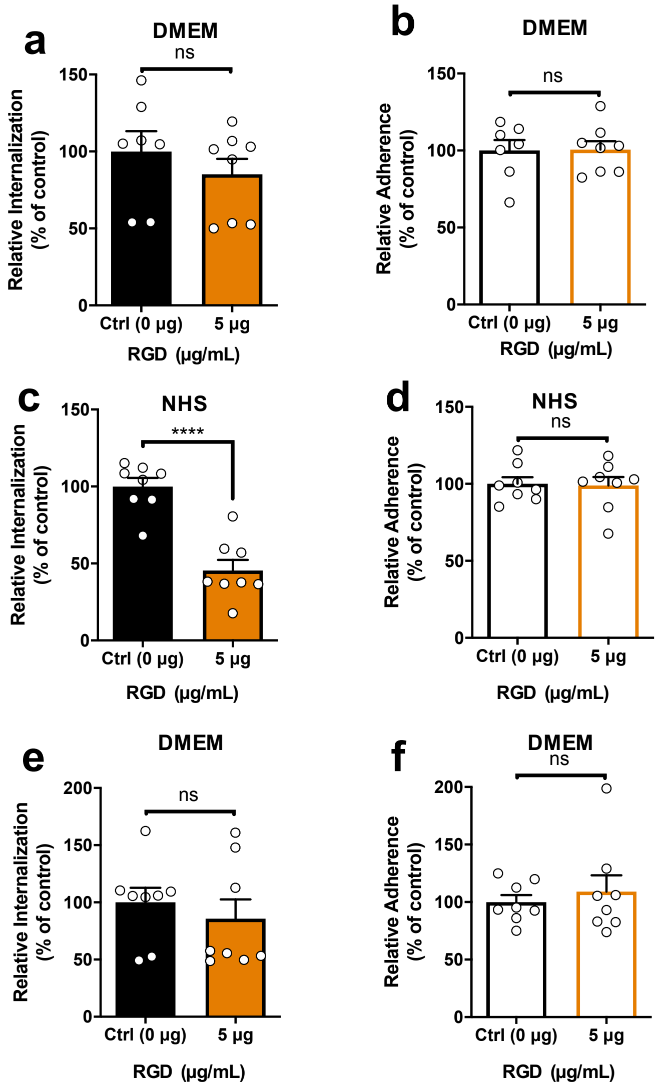

Supplementary Figure 8. The spore entry into Caco-2 cell line in the presence of normal human serum is inhibited by RGD peptide.

Adherence and internalization of *C. difficile* spores in Caco-2 cells that were incubated with RGD peptide. **a** Relative internalization and **b** adherence of *C. difficile* spores preincubated with DMEM in differentiated Caco-2 cells and RGD. **c** Relative internalization and **d** adherence of *C. difficile* spores preincubated with NHS in undifferentiated Caco-2 cells and RGD. **e** Relative internalization and adherence of *C. difficile* spores preincubated with DMEM in undifferentiated Caco-2 cells; treated with RGD. In bars, each dot represents one independent well from three independent experiments. Error bars indicate the mean ± S.E.M. Statistical analysis was performed by two-tailed unpaired Student's *t*-test, ns $p > 0.05$; ****$p < 0.0001$.

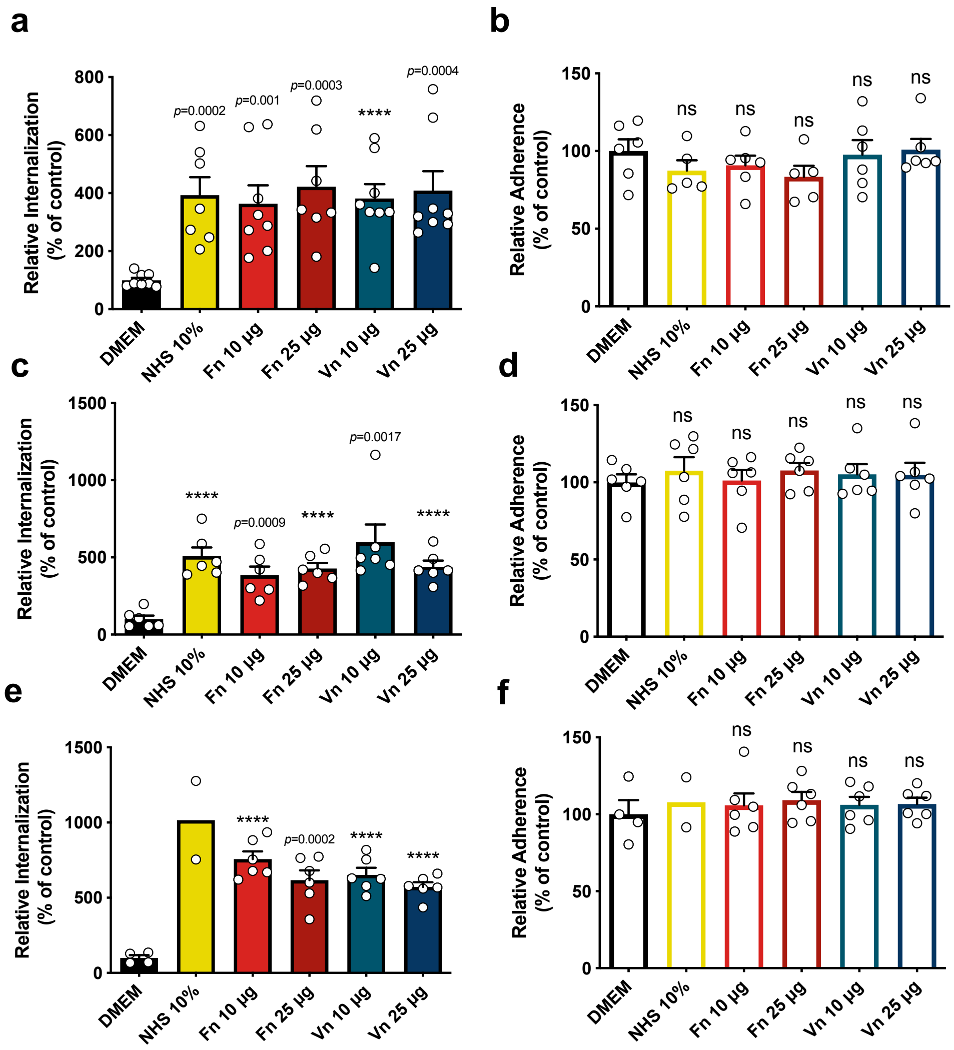

Supplementary Figure 9. *C. difficile* spore entry of spores pre-incubated Fn and Vn and Caco-2 cells pre-incubated Fn and Vn.

Adherence and internalization of *C. difficile* spores in Caco-2 cells in where cells, or spores were incubated with NHS, Fn, or Vn. **a** Relative internalization and **b** adherence of undifferentiated Caco-2 cells infected with *C. difficile* spores pre-treated with NHS, Fn or Vn. **c** Relative internalization and **d** adherence of undifferentiated Caco-2 cells pre-treated with NHS, Fn, or Vn and subsequently infected with *C. difficile* R20291 spores. **e** Relative internalization and **f** adherence of differentiated Caco-2 cells that were pre-treated with NHS, Fn or Vn, and subsequently infected with *C. difficile* R20291 spores. In bars, each dot represents one independent well from three independent experiments. Error bars indicate the mean ± S.E.M. Statistical analysis was performed by two-tailed unpaired Student's *t*-test, ns, $p > 0.05$; ****$p < 0.0001$.

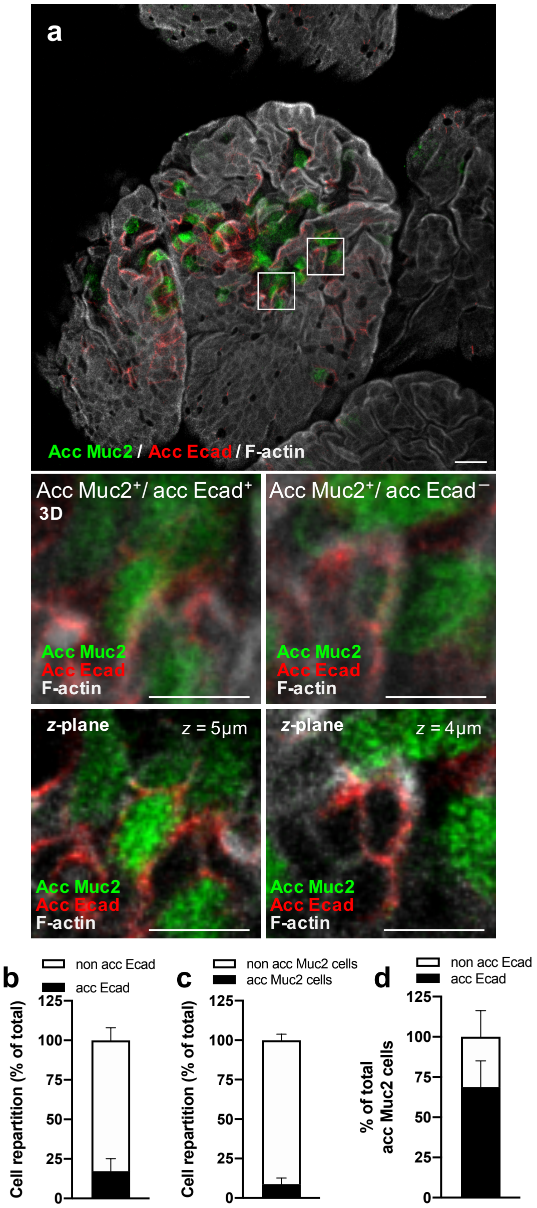

Supplementary Figure 10. Accessibility Muc2 in ileum mucosa. a Confocal micrograph of fixed whole-mounted healthy ileum of mice for accessible Muc2 (acc Muc2) with accessible E-cadherin (acc Ecad). The main figure shown a 3D projection, below magnifications and a z-stack of representative cells with different immunostaining. Repartition of cells immunodetected for **b** for acc Ecad, **c** for Muc2 and **d** total acc Muc2 cells that were immunodetected for acc Ecad is shown. Acc Muc2 is shown in green, acc Ecad is shown in red and F-actin in grey (fluorophores colors were digitally reassigned for a better representation). Scale bar, 20µm, in magnifications 10µm. Micrographs are representative of mice (n = 2). 1,000 - 1,200 cells were counted for each mouse in an area of 84,628µm^2. Error bars indicate mean ± S.E.M.

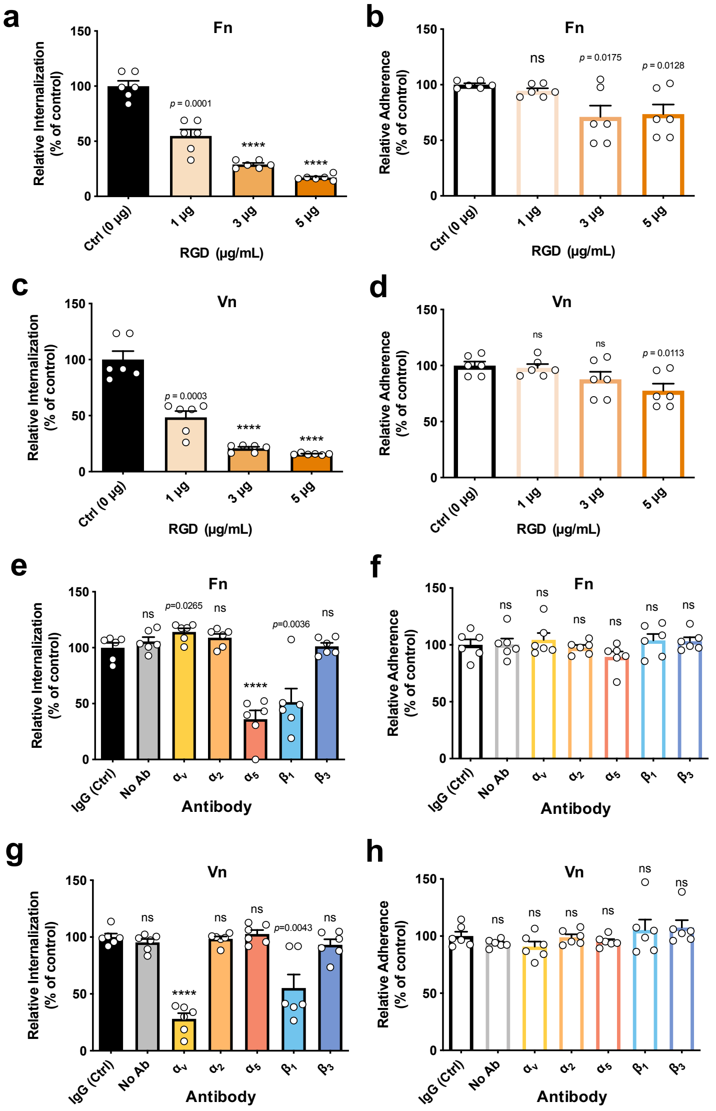

Supplementary Figure 11. The spore entry into Caco-2 cells mediated by Fn occurs through integrin subunits α_5 and β_1; while internalization mediated Vn is through integrin subunits α_v and β_1. a–h undifferentiated and Caco-2 cell monolayer were pre-incubated with **a–d** RGD peptide or **e–h** of antibody against α_v, α_2, α_5, β_1, β_3, non-immune IgG antibody in DMEM and infected with *C. difficile* R20291 spores pre-incubated with **a**, **b**, **e**, **f** Fn, or **c**, **d**, **g**, **h** Vn. **a**, **c**, **e**, **g** Show relative internalization while **b**, **d**, **f**, **h** relative adherence compared to the control. In bars, each dot represents one independent well from three independent experiments. Error bars indicate the mean ± S.E.M. Statistical analysis was performed by two-tailed unpaired Student's *t*-test, ns, $p > 0.05$, ****$p < 0.0001$.

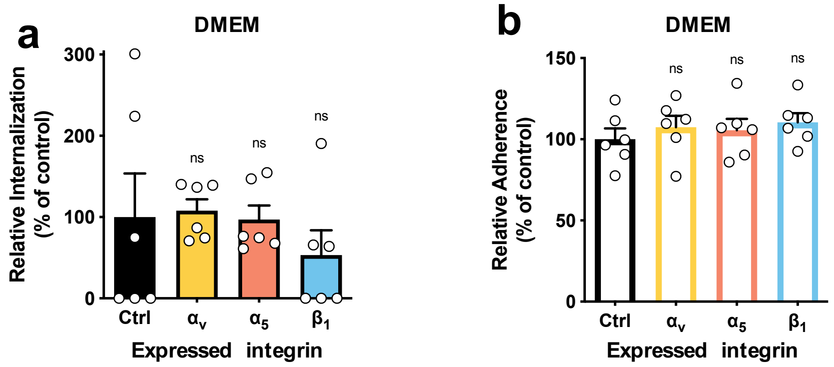

Supplementary Figure 12. Serum-free medium does not promote internalization of *C. difficile* spores dependent of integrins α_v, α_5, and β_1. a Internalization or **b** adherence of *C. difficile* pre-treated 1 h with DMEM in CHO cells ectopically expressing α_v, α_5, β_1 integrins relative to the control (wild-type CHO cells). In bars, each dot represents one independent well from three independent experiments. Error bars indicate the mean ± S.E.M. Statistical analysis was performed by two-tailed unpaired Student's *t*-test, ns, $p > 0.05$.

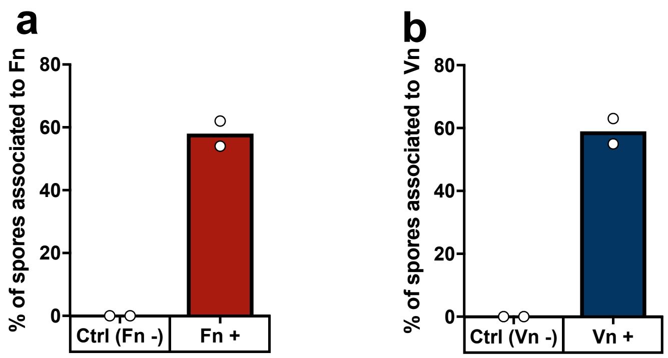

Supplementary Figure 13. Quantification of *C. difficile* spores associated with Fn and Vn by TEM. **a**, **b**, percentage of the total of labeled spores treated with Fn or Vn (Fn + or Vn + respectively) and the negative control without protein (Fn - or Vn - as appropriate). In bars, each dot represents the average of 100 individual spores from two independent experiments.

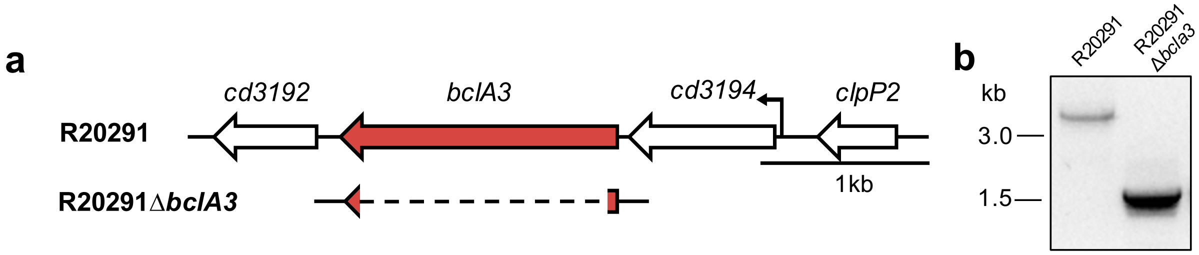

Supplementary Figure 14. Deletion of *bclA3* genes in R20291. The deletion of *bclA3* was done by allelic exchange through a schematic representation of the deletion of *bclA3,* leaving a small peptide in frame. Construction and characterization of Δ*bclA3* in *C. difficile*. **a** Schematic representation of in-frame deletion of *bclA3.* **b** The size of *bclA3* loci was verified by PCR using detection primers. **b**, Is representative PCR for one Δ*bclA3* mutant clone from a total of 10.

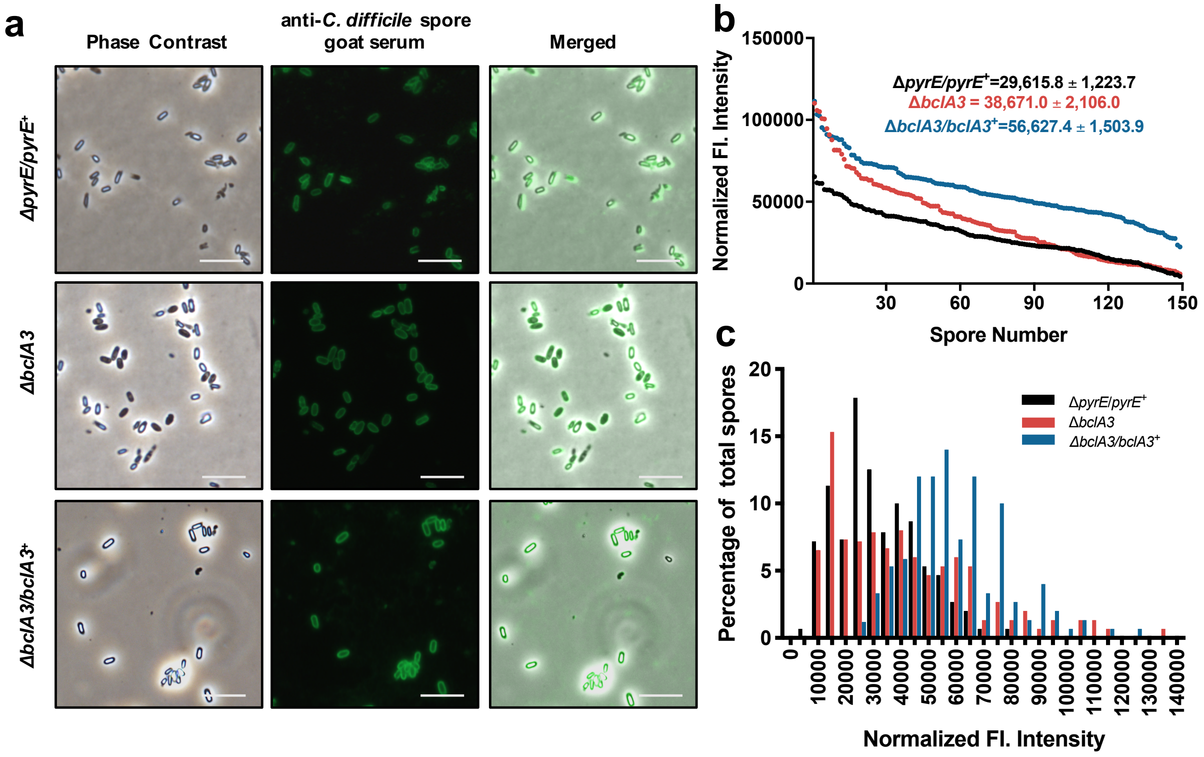

Supplementary Figure 15. Immunofluorescence intensity of the anti-spore in *bclA3* mutant *C. difficile* spores. a Wild-type (Δ*pyrE/pyrE*$^+$), Δ*bclA3* and Δ*bclA3/bclA3*$^+$ R20291 spores are recognized by anti-*C. difficile* spore goat serum. Spores were labeled for 1 h with goat-anti-spore serum and incubated with secondary antibody anti-goat CFL 488-conjugated; then, micrograph were captured with epifluorescence microscopy. **b** Quantitative analysis of the fluorescence (Fl.) intensity in spores of Δ*pyrE/pyrE*$^+$ (dark line), Δ*bclA3* (red line) and Δ*bclA3/bclA3*$^+$ (blue line), the values shown in the graphs the normalized fluorescence intensity from 149 spores of Δ*pyrE/pyrE*$^+$, Δ*bclA3* and Δ*bclA3/bclA3*$^+$ strain. **c** Distribution of the fluorescence intensity of wild-type (dark bars), Δ*bclA3* (red bars) and Δ*bclA3/bclA3*$^+$ (blue bars) spores. Scale bar 10µm.

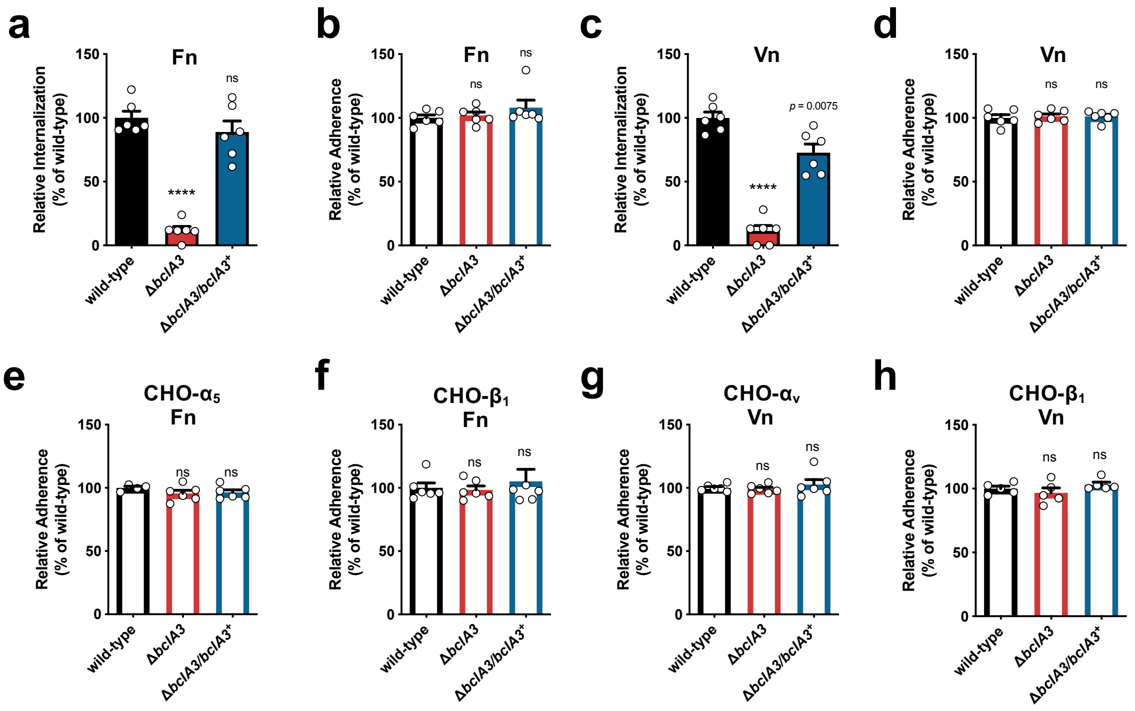

Supplementary Figure 16. Effect of absence of BclA3 on spore entry and adherence to non-phagocytic cells. Relative *C. difficile* spore **a**, **c** internalization and **b**, **d** adherence of HeLa cells infected with *C. difficile* spores Δ*pyrE/pyrE*$^{+}$ (wild-type), Δ*bclA3*, and Δ*bclA3/bclA3*$^{+}$ pre-incubated with **a**, **b** Fn or **c**, **d** Vn. **e**–**h** shown the relative *C. difficile* spore adherence of CHO cells ectopically expressing integrins; **e**, CHO-α_5, **f**, **h** CHO-β_1 and **g** CHO-α_v and infected with *C. difficile* spores wild-type, Δ*bclA3*, and Δ*bclA3/bclA3*$^{+}$ pre-incubated with of **e**, **f** Fn or **g**, **h** Vn. In bars, each dot represents one independent well from three independent experiments. Error bars indicate the mean ± S.E.M. Statistical analysis was performed by two-tailed unpaired Student's *t*-test; ns, $p > 0.05$; ****, $p < 0.0001$.

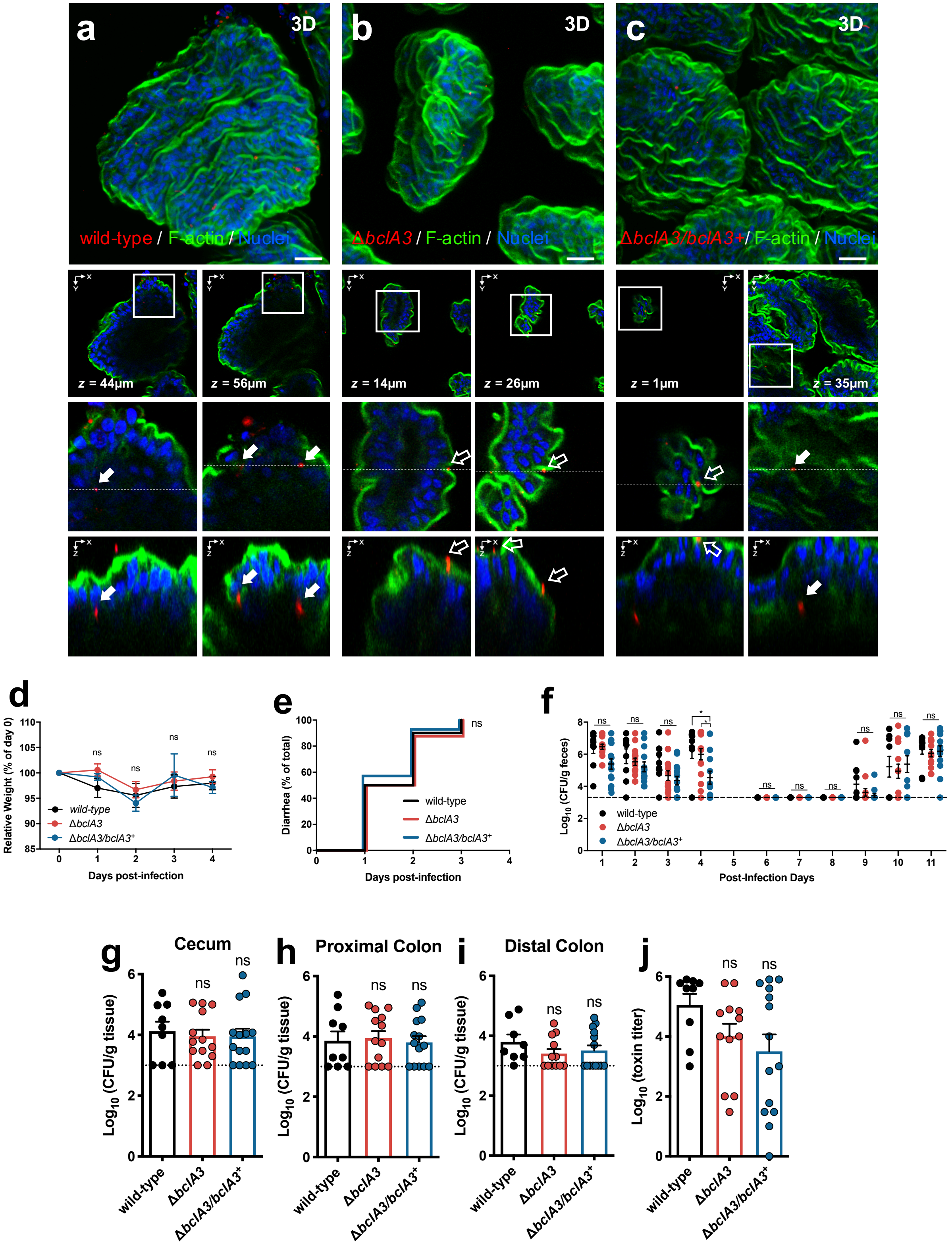

Supplementary Figure 17. Representative confocal micrograph of adhered and internalized *C. difficile* spores in colonic mucosa with Δ*bclA3* spores and Extended data of BclA exosporium protein

contributes to the recurrence of *C. difficile* infection. There are supplementary figures of the experiment detailed in Fig 6. Confocal micrograph of the whole-mount fixed colon. Mice colonic loop model infected with 5 × 10^8 *C. difficile* spores. Panels shown confocal micrographs of adhered and internalized spores in colon loop infected with strains **a** wild-type, **b** Δ*bclA3,* and **c** Δ*bclA3/bclA3*$^+$. The main figure shown a 3D projection below the z-stack, magnification, and the orthogonal view. *C. difficile* spores are shown in red, F-actin is shown in green, and nuclei in blue (fluorophores colors were digitally reassigned for a better representation). The white arrow and empty arrow denote internalized *C. difficile* spores, respectively. Micrograph are representative of intestinal loops infected with *C. difficile* wild type (n = 10); Δ*bclA3* (n = 12) and Δ*pyrE*/*pyrE*$^+$ (n = 11). **d-j** These are supplementary figures from the experiment of R-CDI in animals infected with Δ*bclA3* spores (Fig. 6). Animals were infected with wild-type (n = 10), Δ*bclA3* (n = 16) and Δ*bclA3*/*bclA3*$^+$ (n = 14) and were monitored daily for **d** relative weight; **e** time to diarrhea during CDI. **f** *C. difficile* spore CFU in feces during CDI and R-CDI. Spore adherence to **g** Cecum, **h** proximal colon, **i** distal colon and, **j** cytotoxicity of the cecal was evaluated on day 11. In bars, each dot represents one independent mouse. Error bars indicate the mean ± S.E.M. Statistical analysis was performed by **d**, **f,** Kruskal- Wallis, post-Dunn's test, **e,** Log-rank (Mantel-Cox), **g–j,** two-tailed Mann-Whitney test. ns, $p > 0.05$; *, $p < 0.05$.

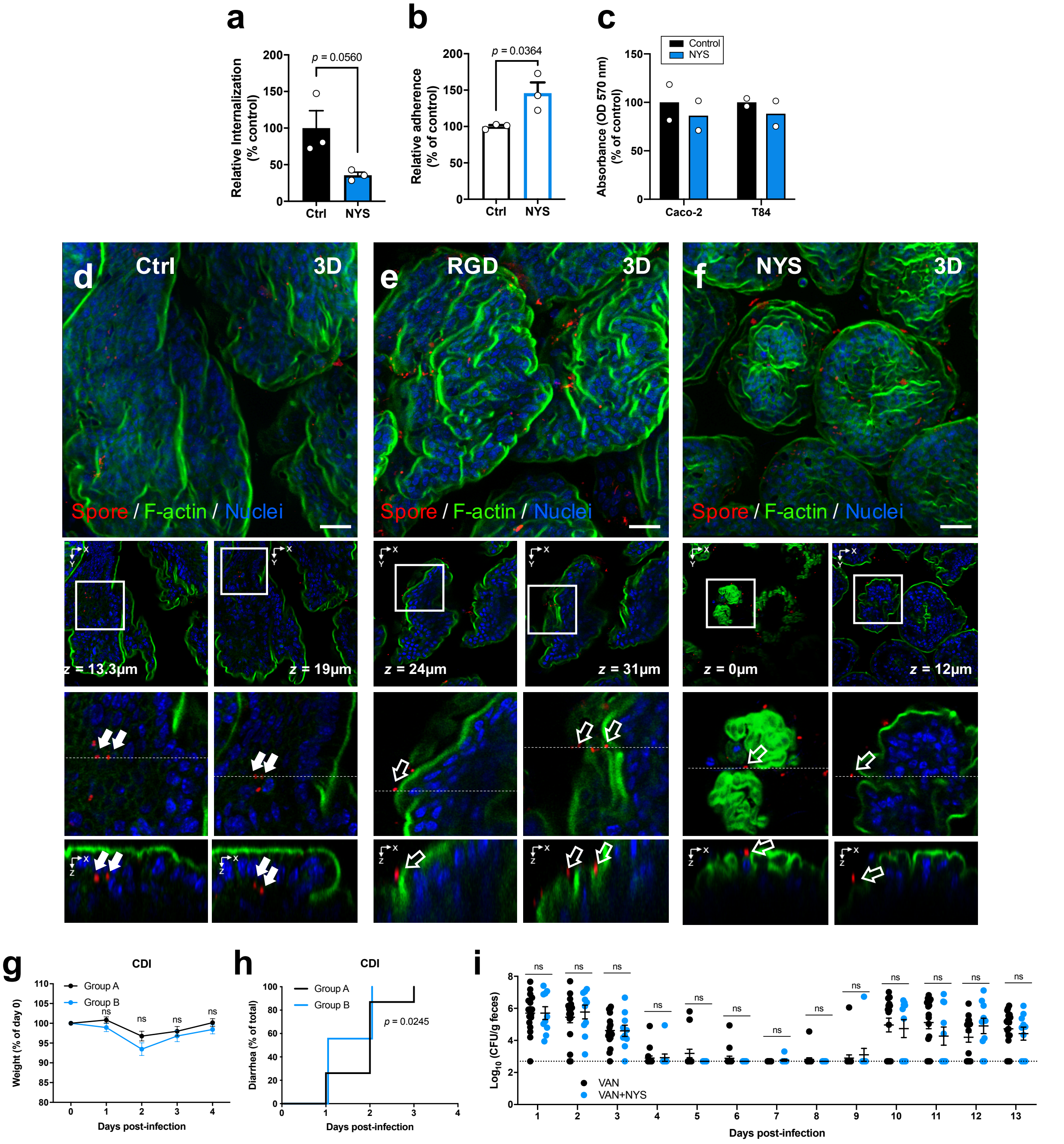

Supplementary Figure 18. Extended data of Fig 7. These are supplementary figures from the experiment detailed in Fig 7. *C. difficile* spore **a** internalization and **b** adherence in monolayers of T84; cells were pre-treated with nystatin (NYS) for 1 h and were subsequently infected with *C. difficile* spores R20291 pre-treated for with FBS. **c** Cellular viability after nystatin treatment was determined by using MTT assay. **d–f** Confocal micrograph of the whole-mount fixed colon. C57BL/6 mice infected in a colonic loop model with 5×10^8 *C. difficile* R20291spores. In bars, each dot represents one independent well. Panels shown adhered

and internalized spores of **d** untreated mice (Ctrl *n*=4), **e** mice treated 24h before the surgery with nystatin, and injected in the intestinal loop in the presence of NYS (*n*=4) and **f** intestinal loop of mice that were injected with RGD peptide in the loop (*n*=4). The main figure shown a 3D projection below the z-stack, magnification, and the orthogonal view. *C. difficile* spores are shown in red, F-actin is shown in green, and nuclei in blue (fluorophores colors were digitally reassigned for a better representation). White arrow and empty arrow denote adhered and internalized *C. difficile* spores, respectively. Micrographs are representative of mice (*n*=4). **g-i** These are supplementary figures from the experiment of R-CDI with animals treated with nystatin and RGD. Animals were infected with *C. difficile* R20291 and were monitored daily for **g** weight loss, **h** diarrhea, and **i** *C. difficile* spore CFU in feces during CDI and R-CDI. For mice treated with nystatin and vancomycin (n = 18) or vancomycin alone as control (n = 23) from days 3-7. Vancomycin is shown as VAN. Error bars indicate the mean ± S.E.M. Statistical analysis was performed by **a, b,** two-tailed unpaired Student's *t*-test, **g, i** two-tailed Mann Whitney test, **h** Log-rank (Mantel-Cox); ns, $p > 0.05$. Scale bar, 20µm.

Supplementary Tables

Supplementary table 1. Bacterial strains and plasmids used

Strain or Plasmid	Relevant characteristic	Source/Reference
E. coli Top10	*mcrA* Δ(*mrr-hsdRMS-mrcBC*) *recA1*	Invitrogen
E. coli CA434	*hsd20*(rB-, mB-, *recA13*, *rpsL20*, *leu*, *proA2*, with IncPb conjugative plasmid R702	[1]
C. difficile 630Δ*erm*	A laboratory strain with erythromycin sensitive derivative of *C. difficile* strain 630	[2]
C. difficile R20291	Ribotype 027, epidemically relevant strain	[3]
C. difficile M120	Ribotype 078 commonly isolated from farm animals	[4]
C. difficile PUC52	Ribotype 027, isolated from a patient with CDI<br>Genome not available	[5,6]
C. difficile PUC30	Ribotype 027, isolated from a patient with CDI<br>Genome not available	[5,6]
C. difficile PUC25	Ribotype 014/020, isolated from a patient with CDI<br>Genome not available	[5,6]
C. difficile PUC31	Ribotype 046, isolated from a patient with CDI<br>Genome not available	[5,6]
C. difficile PUC98	Ribotype 082, isolated from a patient with CDI [-]<br>Genome not available	[5,6]
C. difficile PUC131	Ribotype 097, isolated from a patient with CDI [-]<br>Genome not available	[5,6]
R20291 Δ*pyrE*	R20291 isogenic *pyrE* mutant	[7,8]
R20291 Δ*pyrE/pyrE+*	R20291 isogenic *pyrE* mutant complemented with wild-type *pyrE* into the *pyrE* loci	This work
R20291 Δ*bclA3*	R20291 isogenic *bclA3* mutant	This work
R20291 Δ*bclA3/bclA3+*	R20291 isogenic *bclA3* mutant complemented with wild-type *bclA3* in the *pyrE* loci	This work
Plasmids		

pMTL-YN4	Pseudo suicide vector containing: *Clostridium perfringens catP* resistant cassette, *Clostridium sporogenes pyrE;* Carries unaltered *colE1 E. coli* replicon; *traJ* encoding transfer function of the RP4 *oriT* region; RepA and Orf2, the replication region of the *Clostridium botulinum* plasmid pBP1; and AscI/Sbf1 sites for the cloning of the right-hand homology arm/left-hand homology arm cassette.	[7,8]
pMTL-YN2C	Pseudo suicide vector containing: *Clostridium perfringens catP* cassette; *Clostridium sporogenes pyrE;* Carries unaltered *colE1 E. coli* replicon; *traJ* encoding transfer function of the RP4 *oriT* region; RepA and Orf2, the replication region of the *Clostridium botulinum* plasmid pBP1; a left-hand homology arm encompassing a 300 bp internal fragment of the R20291 *pyrE* gene lacking 50 nucleotides from the 5′-end, and 235 bp from the 3′-end; a right-hand homology arm compromising the 1200 bp region of DNA immediately downstream of *pyrE*; this plasmid also has additional DNA segments inserted between the left-hand and right-hand homology arms which carries a copy of the *lacZ′* containing a multiple cloning site region and a transcriptional terminator of the ferredoxin gene.	[7,8]
pDP376	A 1086 bp PCR fragment was made by overlap extension PCR of a 544pb LHA upstream of the start codon of *bclA3* and a 542 bp RHA downstream of *bclA3* of strain R20291 The cassette was cloned by Gibson assembly into AscI/SbfI digested pMTL-YN4 vector.	This work
pMPG1	A 3565-bp fragment containing the promoter region of the entire bicistronic operon formed by *sgtA* and *bclA3* of *C. difficile* strain R20291 was cloned into EcoRI and BamHI sites of pMTL-YN2C. Plasmid to complement the bclA3 mutant strain in the *pyrE* loci.	This work

Supplementary table 2. Primers used

Primer code/Name	Primer sequence[a]	Position[b]	Gene	Use[c]
P332<br>FP-LHA-bclA3- *pyrE*	*CCGATCGGGCCC*CCTGCAGGTGCTAGGTATAAAGA<br>AGCAATAGAAGG	-544 to -517	*bclA3*	MP
P334<br>RP-LHA-bclA3-pyrE	*CGATATTAGAAGCCTTTTCTTATCTAATCTA*TATTAAAA<br>GCACCTCCTGATATATTTGAGC	-30 to -1	*bclA3*	MP
P335<br>FP-RHA-bclA3-pyrE	*GCTCAAATATATCAGGAGGTGCTTTTAATA*TAGATTAG<br>ATAAGAAAAGGCTTCTAATATCG	+2034 to +2065	*bclA3*	MP
P336<br>RP-RHA-b*clA3*-pyrE	*GCTAAGGATTCAGAAC*GGCGCGCCCCAGGAATAGCATATGAAT<br>TAGCCC	+2551 to +2576	*bclA3*	MP
P664<br>FP-bclA3-detect	GAGAGGTATTTTATTCTGACATTGC	-699 to -673	*bclA3*	MP
P665<br>RP-bclA3-detect	GCTTCCTATTCTTCCACAACACC	+2689 to +2712	*bclA3*	MP
P476<br>NFP-bclA3c-promotor	*CCATGATTAC*GAATTCGAGGCTAAAGAGTACGGGCT<br>GATTG	-1527 to -1502	*bclA3*	CP
P477<br>NRP-bclA3c	*GACTCTAGA*GGATCCCTAATTTATTGCAATTCCTGCA<br>CTTGCATATCC	+2004 to +2037	*bclA3*	CP
P530<br>FP-pyrE detect	AGAGAAGGAATAAAAAGTTTAGACGAAATAAGAG<br>G			CP
P529<br>RP-pyrE detect	TTACATCCCTAATTCCTTGAACTCTC			CP

[a] Restriction sites are underlined; added sequence for proper restriction digestion are in italic; overlapping sequence for overlap extension PCR or Gibson cloning are in italic and grey.

[b] The nucleotide position numbering begins from the first codon and refers to the relevant position within the respective gene sequence. Restriction sites EcoRI (GAATTC), BamHI (GGATCC), Sbf1 (CCTGCAGG, AscI (GGCGCGCC) are underlined.

[c] MP, deletion of target gene; CP, complementation of target gene.

Supplementary References

1 Emerson, J. E. *et al*. A novel genetic switch controls phase variable expression of CwpV, a Clostridium difficile cell wall protein. *Mol Microbiol* **74**, 541-556.

2 Hussain, H. A., Roberts, A. P. & Mullany, P. Generation of an erythromycin-sensitive derivative of Clostridium difficile strain 630 (630Deltaerm) and demonstration that the conjugative transposon Tn916DeltaE enters the genome of this strain at multiple sites. *J Med Microbiol* **54**, 137-141.

3 McEllistrem, M. C., Carman, R. J., Gerding, D. N., Genheimer, C. W. & Zheng, L. A hospital outbreak of Clostridium difficile disease associated with isolates carrying binary toxin genes. *Clin Infect Dis* **40**, 265-272.

4 Goorhuis, A. *et al*. Emergence of Clostridium difficile infection due to a new hypervirulent strain, polymerase chain reaction ribotype 078. *Clin Infect Dis* **47**, 1162-1170.

5 Plaza-Garrido, A. *et al*. Predominance of *Clostridium difficile* ribotypes 012, 027 and 046 in a university hospital in Chile, 2012. *Epidemiol Infect* **144**, 976-979.

6 Plaza-Garrido, A. *et al*. Outcome of relapsing Clostridium difficile infections do not correlate with virulence-, spore- and vegetative cell-associated phenotypes. *Anaerobe* **36**, 30-38.

7 Ehsaan, M., Kuehne, S. A. & Minton, N. P. *Clostridium difficile* Genome Editing Using *pyrE* Alleles. *Methods Mol Biol* **1476**, 35-52.

8 Ng, Y. K. *et al*. Expanding the repertoire of gene tools for precise manipulation of the *Clostridium difficile* genome: allelic exchange using *pyrE* alleles. *PLoS One* **8**, e56051.
