# Supplementary material for: Entry of spores into intestinal epithelial cells contributes to recurrence of Clostridioides difficile infection
Source: Nat Commun. 2021 Feb 18;12:1140. doi: 10.1038/s41467-021-21355-5 (PMC7893008; doi:10.1038/s41467-021-21355-5)
Supplement: Supplementary file 3 — Description of Additional Supplementary Files [file 41467_2021_21355_MOESM3_ESM.pdf]

Description of Additional Supplementary Files

File Name: Supplementary Movie 1
Description: **Wild-type *C. difficile* spores internalize in small intestine.** Travel through a confocal *Z*-stack from the apical face of the small intestine. F-actin is visualized with fluorescently labeled phalloidin with Alexa-Fluor 488 (green) to distinguish the structure of the intestinal villus (green). And *C. difficile* spores were detected using anti-*C. difficile* chicken spore antibody IgY and goat anti-chicken IgY secondary antibody conjugated with Alexa-Fluor 568 (red). DNA was stained with DAPI (blue). Arrows indicate internalized spores.

File Name: Supplementary Movie 2
Description: **Wild-type *C. difficile* spores internalize in colonic mucosa.** Travel through a confocal *Z*-stack from the apical face of the colonic mucosa. F-actin is visualized with fluorescently labeled phalloidin Alexa-Fluor 488 (green) to distinguish the structure of the colonic crypt, and *C. difficile* spores were detected using anti-*C. difficile* chicken spore antibody IgY and goat anti-chicken IgY secondary antibody conjugated with Alexa-Fluor 568 (red). Arrows indicate internalized spores.
