# Supplementary material for: Entry of spores into intestinal epithelial cells contributes to recurrence of Clostridioides difficile infection
Source: Nat Commun. 2021 Feb 18;12:1140. doi: 10.1038/s41467-021-21355-5 (PMC7893008; doi:10.1038/s41467-021-21355-5)
Supplement: Supplementary file 6 — Reporting Summary [file 41467_2021_21355_MOESM6_ESM.pdf]

nature research

Corresponding author(s): Daniel Paredes-Sabja

Last updated by author(s): Jan 15, 2021

# Reporting Summary

Nature Research wishes to improve the reproducibility of the work that we publish. This form provides structure for consistency and transparency in reporting. For further information on Nature Research policies, see Authors & Referees and the Editorial Policy Checklist .

## Statistics

For all statistical analyses, confirm that the following items are present in the figure legend, table legend, main text, or Methods section.

| n/a | Confirmed | |
|---|---|---|
| ☐ | ☒ | The exact sample size (*n*) for each experimental group/condition, given as a discrete number and unit of measurement |
| ☐ | ☒ | A statement on whether measurements were taken from distinct samples or whether the same sample was measured repeatedly |
| ☐ | ☒ | The statistical test(s) used AND whether they are one- or two-sided<br>*Only common tests should be described solely by name; describe more complex techniques in the Methods section.* |
| ☒ | ☐ | A description of all covariates tested |
| ☐ | ☒ | A description of any assumptions or corrections, such as tests of normality and adjustment for multiple comparisons |
| ☐ | ☒ | A full description of the statistical parameters including central tendency (e.g. means) or other basic estimates (e.g. regression coefficient) AND variation (e.g. standard deviation) or associated estimates of uncertainty (e.g. confidence intervals) |
| ☒ | ☐ | For null hypothesis testing, the test statistic (e.g. *F*, *t*, *r*) with confidence intervals, effect sizes, degrees of freedom and *P* value noted<br>*Give P values as exact values whenever suitable.* |
| ☒ | ☐ | For Bayesian analysis, information on the choice of priors and Markov chain Monte Carlo settings |
| ☒ | ☐ | For hierarchical and complex designs, identification of the appropriate level for tests and full reporting of outcomes |
| ☒ | ☐ | Estimates of effect sizes (e.g. Cohen's *d*, Pearson's *r*), indicating how they were calculated |

*Our web collection on statistics for biologists contains articles on many of the points above.*

## Software and code

Policy information about availability of computer code

| | |
|---|---|
| Data collection | Confocal Imaging was acquired with:<br>Leica TCS LSI (software Leica Application Suite X (LASX) 3.1.2.16221), Life Science (Leica),<br>Leica SP8 (software Leica Application Suite X (LASX) 3.5.5.19976), Life Science (Leica), and<br>Olympus FV1000 (software Olympus Fluoview (FV10-ASW) 04.02.03.06 |
| Data analysis | Microsoft excel v16.43. Confocal images were processed and analyzed using Fiji v1.53g and statistics analysis was done with GraphPad Prism 7.0a. |

For manuscripts utilizing custom algorithms or software that are central to the research but not yet described in published literature, software must be made available to editors/reviewers. We strongly encourage code deposition in a community repository (e.g. GitHub). See the Nature Research guidelines for submitting code & software for further information.

## Data

Policy information about availability of data

All manuscripts must include a data availability statement. This statement should provide the following information, where applicable:

- Accession codes, unique identifiers, or web links for publicly available datasets
- A list of figures that have associated raw data
- A description of any restrictions on data availability

The data that support the findings of this study are available from the corresponding author upon reasonable request. Source data are provided with this paper.

# Field-specific reporting

Please select the one below that is the best fit for your research. If you are not sure, read the appropriate sections before making your selection.

☒ Life sciences ☐ Behavioural & social sciences ☐ Ecological, evolutionary & environmental sciences

For a reference copy of the document with all sections, see nature.com/documents/nr-reporting-summary-flat.pdf

# Life sciences study design

All studies must disclose on these points even when the disclosure is negative.

| | |
|---|---|
| Sample size | Sample size was not predetermined using statistical methods. Sample size was as large as possible with at least two three independent replicates in critical experiment and the number was sufficient to support the statistical analyses performed in this manuscript.<br>For mouse experiments, the luminally accessibility of Fn and Vn was visualized in n=2 mice and and in intestinal ligated loop experiment and R-CDI experiments we used at least n ≥ 4 per experimental condition to reach statistical significance with the minimum number of animals. |
| Data exclusions | No data were excluded from analysis. |
| Replication | The critical experiments in vitro were repeated at least two to three independent experiments with similar results. Number of replication of in vitro and mouse experiments is indicated in each Figure legend |
| Randomization | For cell culture experiments,randomized cell culture wells were used.<br>For animal experiments, mice were randomly assigned to the different groups.<br>epifluorescence and confocal microscopy images were obtained in random fields. |
| Blinding | The investigators were not blinded the development of the animal experiments to avoid cross contamination between animals belonging to different treatment groups or infected with different strains. In the case of R-CDI experiments of mice treated with NYS we ensure the correct administration of the treatments to animals of the different groups.<br>However, for counting of adherence and internalized spores as well the analysis of epifluorescence images and confocal microscopy investigator were blinded. |

# Reporting for specific materials, systems and methods

We require information from authors about some types of materials, experimental systems and methods used in many studies. Here, indicate whether each material, system or method listed is relevant to your study. If you are not sure if a list item applies to your research, read the appropriate section before selecting a response.

## Materials & experimental systems

| n/a | Involved in the study | |
|---|---|---|
| ☐ | ☒ | Antibodies |
| ☐ | ☒ | Eukaryotic cell lines |
| ☒ | ☐ | Palaeontology |
| ☐ | ☒ | Animals and other organisms |
| ☒ | ☐ | Human research participants |
| ☒ | ☐ | Clinical data |

## Methods

| n/a | Involved in the study | |
|---|---|---|
| ☒ | ☐ | ChIP-seq |
| ☒ | ☐ | Flow cytometry |
| ☒ | ☐ | MRI-based neuroimaging |

## Antibodies

Antibodies used

1) Chicken IgY anti-C. difficile spore batch 7246 (Aveslab, USA).
2) Goat Serum anti-C. difficile spore PAC 5573 (Pacific Immunology, USA).
3) Rabbit pAb anti-fibronectin (SC9068, Santa Cruz Biotechnology).
4) Rabbit pAB anti-vitronectin (SC15332, Santa Cruz Biotechnology).
5) Mouse monoclonal anti-human integrin α5 (ab78614 Abcam).
6) Mouse monoclonal anti-human integrin αv (ab16821 Abcam).
7) Mouse monoclonal anti-human integrin α2 (MAB1950Z Millipore).
8) Mouse monoclonal anti-human integrin β1 (MAB1959Z Millipore).
9) Mouse monoclonal anti-human integrin β3 (MAB2023Z Millipore).
10) Mouse anti-integrin αv (SC166665 Santa Cruz Biotechnologies).
11) Mouse anti-integrin α5 (SC376156 Santa Cruz Biotechnologies).
12) Mouse anti-integrin β1( SC374429 Santa Cruz Biotechnologies).
13) Mouse monoclonal anti-α-tubulin (T5168 Sigma-Aldrich).
14) Goat anti-mouse horseradish peroxidase conjugate (A5278, Sigma-Aldrich).

15) Goat anti-chicken IgY secondary antibodies Alexa-Fluor 488 (#ab150173 Abcam).
16) Donkey anti-goat conjugated with CFL 488 secondary antibody (SC362255, Santa Cruz Biotechnologies).
17) chicken anti-rat IgG secondary antibodies Alexa-Fluor 488 (A-21470, ThermoFisher)
18) Rabbit polyclonal anti- human Muc-2 (ab90007, Abcam)
19) Goat polyclonal anti-rabbit IgG secondary antibodies Alexa-Fluor 568 (A11036, Invitrogen)
20) donkey anti-rabbit IgG antibody coupled to 12–nm gold particles (ab105295, Abcam)
21) Rat monoclonal anti-E cadherin (ab11512 Abcam)
22) IgG from rabbit serum (I5006, Sigma-Aldrich).

Validation

1) Chicken IgY anti-C. difficile spore batch 7246 (Aveslab)
Validation: This antibody was previously published in doi: 10.3389/fcimb.2017.00365

2) Goat serum anti-C. difficile spore PAC 5573 (Pacific Immunology)
Validation: This antibody was previously published in doi: 10.1016/j.anaerobe.2013.11.003

3) Rabbit pAb anti-fibronectin (SC9068, Santa Cruz Biotechnology)
Validation: https://www.scbt.com/p/fibronectin-antibody-h-300
Information of the manufacturer website:
* Fibronectin Antibody (H-300) is a rabbit polyclonal IgG; 200 µg/ml
* epitope corresponding to amino acids 2087-2386 mapping at the C-terminus of Fibronectin of human origin
* Product citations (60)

4) Rabbit pAB anti-vitronectin (SC15332, Santa Cruz Biotechnology)
Validation: https://www.scbt.com/p/vitronectin-65-75-antibody-h-270
Information of the manufacturer website:
* Vitronectin 65/75 Antibody (H-270) is a rabbit polyclonal IgG
* epitope corresponding to amino acids 1-270 mapping at the N-terminus of Vitronectin 75 of human origin
* Product citations (6)

5) Mouse monoclonal anti-human integrin α5 (ab78614 Abcam)
Validation: https://www.abcam.com/integrin-alpha-5-antibody-p1d6-ab78614.html
Information of the manufacturer website:
* Mouse monoclonal [P1D6] to Integrin alpha 5
* Positive control FC: HeLa cells, HT-1080 cells
* Reacts with: Human

6) Mouse monoclonal anti-human integrin αv (ab16821 Abcam)
Validation: https://www.abcam.com/integrin-alpha-v-antibody-272-17e6-ab16821.html
Information of the manufacturer website:
* Mouse monoclonal [272-17E6] to Integrin alpha V
* Reacts with Human, Does not react with mouse
* Immunogen: Full length native protein (purified) (Human).
* Positive control: Human breast carcinoma

7) Mouse monoclonal anti-human integrin α2 (MAB1950Z Millipore)
Validation: https://www.emdmillipore.com/US/en/product/Anti-Integrin-2-Antibody-clone-P1E6-azide-free,MM_NF-MAB1950Z
Information of the manufacturer website:
* This Anti-Integrin α2 Antibody, clone P1E6, azide free is validated for use in IC, IH, FUNC for the detection of Integrin α2
* Control Skin (Basement membrane)
* Application Notes: Suitable for use in attachment inhibition assays using fibroblasts, epithelial cells, endothelial cells, and non-activated platelets on collagen types I, III, IV, VI and laminin.

8) Mouse monoclonal anti-human integrin β1 (MAB1959Z Millipore)
Validation: https://www.emdmillipore.com/US/en/product/Anti-Integrin-1-Antibody-clone-P5D2-Azide-Free-MAB1959,MM_NF-MAB1959Z
Information of the manufacturer website:
* Anti-Integrin β1 Antibody, clone P5D2 (Azide Free) detects level of Integrin β1 & has been published & validated for use in ELISA, FC, IC, IH, IP, FUNC.
* Control: Human tonsil, human skin tissue, A431 & HeLa Cells
* Functional Activity Assay: A representative lot of this antibody clone was used in cell attachment assay of SV-HFO cells with a characteristic spread morphology. In the presence of function-blocking mAbs to β1 integrin (P5D2), the cells attached but no longer spread, and displayed a rounded morphology with many cytoplasmic projections (Iba, K. et al., 2000).

9) Mouse monoclonal anti-human integrin β3 (MAB2023Z Millipore)
Validation: https://www.emdmillipore.com/US/en/product/Anti-Integrin-3-Antibody-clone-B3A-azide-free,MM_NF-MAB2023Z

Information of the manufacturer website:
* Anti-Integrin β3 Antibody, clone B3A, azide free detects level of Integrin β3 & has been published & validated for use in FC, FUNC & WB.
* Positive Control: U251 or D54 human cell lines. Membrane preps are recommended for western blots.
Human endothelium cells (expression of integrin alpha V beta 3 may need to be upregulated by phorbol esters stimulation). Skin (Basement Membrane)
* Application Notes Function-blocking of integrin beta3-mediated adhesion to ECM proteins, Western blot (reducing conditions) Optimal working dilutions must be determined by end user.

10) Mouse anti-integrin αv (SC166665 Santa Cruz Biotechnologies)
Validation: https://www.scbt.com/p/integrin-alpha5-antibody-a-11?productCanUrl=integrin-alpha5-antibody-a-11&_requestid=4308836
Information of the manufacturer website:
* Integrin alpha 5 Antibody (A-11) is recommended for detection of Integrin α5 of mouse, rat and human origin by WB, IP, IF, IHC (P) and ELISA.
* raised against amino acids 840-943 of Integrin α5 of human origin
* product citations (6)

11) Mouse anti-integrin α5 (SC376156 Santa Cruz Biotechnologies)
Validation: https://www.scbt.com/p/integrin-alphav-antibody-h-2
Information of the manufacturer website:
* specific for an epitope mapping between amino acids 859-888 at the C-terminus of Integrin αV of human origin
* Integrin alpha V Antibody (H-2) is recommended for detection of Integrin αV heavy chain of mouse, rat and human origin by WB, IP, IF and ELISA
* Product citations (10)

12) Mouse anti-integrin β1(SC374429 Santa Cruz Biotechnologies)
Validation: https://www.scbt.com/p/integrin-beta1-antibody-a-4
Information of the manufacturer website:
* raised against amino acids 375-480 mapping within an extracellular domain of Integrin β1 of human origin
* Integrin beta 1 Antibody (A-4) is recommended for detection of Integrin β1 of mouse, rat and human origin by WB, IP, IF and ELISA
* Product citations (22)

13) Mouse monoclonal anti-α-tubulin (T5168 Sigma-Aldrich)
Validation: https://www.sigmaaldrich.com/catalog/product/sigma/t5168?lang=en&region=US
* Monoclonal Anti-α-Tubulin (mouse IgG1 isotype) is derived from the B-5-1-2 hybridoma produced by the fusion of mouse myeloma cells and splenocytes from an immunized mouse. Recognizes an epitope located at the C-terminal end of the α-tubulin isoform in a variety of organisms.
* Application In western blotting/ Immunoblotting
* Product Citation (1904)

14) Goat anti-mouse horseradish peroxidase conjugate (A5278, Sigma-Aldrich).
Validation: https://www.sigmaaldrich.com/catalog/product/sigma/a5278?lang=es&region=CL
* Product citations 72.

15) Goat anti-chicken IgY secondary antibodies Alexa-Fluor 488 (#ab150173 Abcam).
Validation: https://www.abcam.com/goat-chicken-igy-hl-alexa-fluor-488-preadsorbed-ab150173.html
* Product citations 20.

16) Donkey anti-goat conjugated with CFL 488 secondary antibody (SC362255, Santa Cruz Biotechnologies).
Validation: https://www.scbt.com/p/donkey-anti-goat-igg-cfl-488

17.- chicken anti-rat IgG secondary antibodies Alexa-Fluor 488 (A-21470, ThermoFisher)
Validation: https://www.thermofisher.com/antibody/product/Chicken-anti-Rat-IgG-H-L-Cross-Adsorbed-Secondary-Antibody-Polyclonal/A-21470
This antibody was previously published in doi: 10.1016/j.stemcr.2017.11.021.

18.- Rabbit polyclonal anti- human Muc-2 (ab90007, Abcam)
Validation: This antibody was previously published in doi: 10.1158/0008-5472.CAN-17-3487

19.- Goat polyclonal anti-rabbit IgG secondary antibodies Alexa-Fluor 568 (A11036, Invitrogen)
Validation: https://www.thermofisher.com/antibody/product/Goat-anti-Rabbit-IgG-H-L-Highly-Cross-Adsorbed-Secondary-Antibody-Polyclonal/A-11036

* Product citations: 196

20.- donkey anti-rabbit IgG antibody coupled to 12–nm gold particles (ab105295, Abcam)
Validation: https://www.abcam.com/donkey-rabbit-igg-hl-12nm-gold-preadsorbed-ab105295.html
Information of the manufacturer website
* Donkey Anti-Rabbit IgG H&L (12nm Gold) preadsorbed
* Conjugation: Gold 12nm
* Host species: Donkey
* Suitable for: Electron Microscopy, ELISA, WB

21.- Rat monoclonal anti-E cadherin (ab11512 Abcam)
Validation: https://www.abcam.com/e-cadherin-antibody-decma-1-intercellular-junction-marker-ab11512.html
* Product citations 89.

22.- IgG from rabbit serum (I5006, Sigma-Aldrich).
Validation: https://www.sigmaaldrich.com/catalog/product/sigma/i5006?lang=en&region=US
* Product citations 77.

# Eukaryotic cell lines

Policy information about cell lines

| | |
|---|---|
| Cell line source(s) | The human colorectal adenocarcinoma Caco-2 cells were obtained from ATCC (ATCC HTB-37).<br>The African green monkey kidney cells Vero-E6 were obtained from ATCC (ATCC CCL-81).<br>The human cervix epithelial cells HeLa were obtained from ATCC (ATCC CCL-2).<br>The human colorectal carcinoma T84 cells were provided by Mauricio Farfán (Universidad de Chile).<br>The Chinese hamster ovary cells CHO-k1 were obtained from ATCC (ATCC CCL-61)<br>The human colorectal adenocarcinoma HT29 cells were obtained from ATCC (ATCC HTB-38). |
| Authentication | None of the cells lines used have been authenticated |
| Mycoplasma contamination | Yes, Mycoplasma contamination was assessed, and were negative throughout the course of these studies. |
| Commonly misidentified lines<br>(See ICLAC register) | No misidentified cells lines were used |

# Animals and other organisms

Policy information about studies involving animals; ARRIVE guidelines recommended for reporting animal research

| | |
|---|---|
| Laboratory animals | Mice were housed with ad libitum access to food and water. Bedding and cages were autoclaved, and mice had a 12-hour cycle of light and darkness. Mice were housed at 20–24C with 40–60% of humidity. All procedures complied with all relevant ethical regulations for animal testing and research. This study received ethical approval by the Institutional Animal Care and Use Committee of the Universidad Andrés Bello. |
| Wild animals | Wild animals were not used in this study |
| Field-collected samples | No field collected samples were used in the study. |
| Ethics oversight | All procedures were performed following the approved protocols by the Institutional Animal Care and Use Committee of the Universidad Andrés Bello. |

Note that full information on the approval of the study protocol must also be provided in the manuscript.
